# Supplementary material for: Are carbon emissions trading and green financial instruments synergistic? -Comprehensive quantitative research based on content analysis
Source: PLoS One. 2024 Mar 7;19(3):e0298601. doi: 10.1371/journal.pone.0298601 (PMC10919601; doi:10.1371/journal.pone.0298601)
Supplement: S1 Data — (ZIP) [file pone.0298601.s001.zip › Data/地级市Z_F工作报告.zip!/╦─┤¿/╣π░▓╩╨/└┤╫╘╣1⁄2╓┌║┼í╛┬φ┐╦╩2╛▌═°í┐_2015.docx]

各位代表：

我代表市人民政府，向大会报告过去一年的政府工作，对今年工作提出建议，请各位代表审查，并请市政协委员提出意见。

2014年工作回顾

过去一年，是全面深化改革的开局之年，也是广安攻坚破难卓有成效的一年。在市委的坚强领导下，在市人大和市政协的监督支持下，我们认真贯彻落实党中央、国务院、省委、省政府和市委的决策部署，始终保持专注发展定力，深入实施“三大发展战略”，统筹稳增长、调结构、促改革、惠民生，全市经济快速发展、民生持续改善、社会和谐稳定。主要经济指标增速位居全省前列，全年实现地区生产总值919.6亿元、增长10.2%，增速居全省第2位；全社会固定资产投资、服务业增加值增速均居全省第1位，地方一般公共预算收入、城乡居民收入增速均居全省第5位，社会消费品零售总额、规上工业增加值增速均居全省第8位。

一年来，主要做了以下工作。

成功筹办邓小平同志诞辰110周年纪念活动。按照隆重、热烈、简朴、务实原则，精心筹办邓小平同志诞辰110周年纪念活动，掀起了缅怀小平、感恩奋进的热潮，加快了广安发展，惠及了广大群众。统筹建设主体纪念设施。全面完成邓小平缅怀馆建设和邓小平故居陈列馆改陈工作，恢复改造翰林院子等小平同志青少年时期活动场所，进一步丰富了邓小平故里爱国主义教育基地内涵。精心组织主题纪念活动。成功承办省委在广安召开的纪念座谈会，积极协调中国文联文艺志愿服务团、中央电视台“心连心”艺术团先后到广安开展慰问演出，与人民网共同建设邓小平纪念网，与中国邮政总公司共同发行了邓小平同志诞生110周年纪念邮票，参与并承办了在香港、澳门举办的“光辉历程--纪念邓小平诞辰110周年大型展览”，在中国美术馆举行了《春天的故事--全国版画艺术精品展》，举办了《感恩小平·中国梦在广安》大型文艺晚会，组织开展广场音乐诗会、焰火晚会、全民健身跑等大规模群众纪念活动，各项活动的成功举办，营造了缅怀小平的浓厚氛围。广泛开展感恩作贡献活动。组织开展“感恩小平·我为小平家乡发展作贡献”活动，动员社会各界参与“感恩小平·共建广安”活动，争取中宣部、国家发改委、财政部等部委“两馆”建设布展资金1.7亿元，衔接落实兄弟市州捐赠资金1.9亿元、驻川部队捐赠1300万元、社会捐赠3.4亿元；规划实施了市民广场及文化综合体等36个重大民生项目，在全社会凝聚起了感恩小平共促发展的强大合力。

综合施策稳定经济增长。积极应对宏观经济下行压力和传统骨干产业结构性萎缩的影响，研究出台20条稳增长措施及一系列配套政策，及时解决经济运行中的突出矛盾和问题，从多方面稳定经济增势。充分发挥投资对稳增长的关键作用。突出抓好基础设施、节能环保、民生保障等领域投资，重点实施一批夯基础、补短板、增后劲、惠民生的重大项目，417个投资1000万元以上项目完成投资575.8亿元，长明高新技术产业园、玖源化工窑法磷酸、希施凯手机整机生产等项目开工建设，巴广渝高速及遂广高速广安段、宏威新材料等项目加快建设，玖源化工一期、诚信化工二期、临港大市场等项目竣工投产，兰渝铁路广安支线全面建成，广安至成都开通动车，广安进入动车时代。全社会固定资产投资总额达919.2亿元、增长36.7%。着力发挥消费对稳增长的基础作用。加强城乡消费基础设施建设，完善城乡市场体系，摩尔春天、华润万家、欧丹百货、岳池东湖城市综合体等投入营运，枣山现代粮食物流园区、农产品批发市场一期建成，武胜、邻水农产品产地批发市场建设有序推进；积极参加全川市场拓展“三大活动”，组织开展名优特产、汽车、家居建材等各类展销会，培育了一批消费热点，实现社会消费品零售总额330.4亿元、增长13.1%。积极发挥工业对稳增长的主体作用。针对年初工业增速低迷状况，实施“一企一策”，加强分类指导，开展“入企结对帮扶稳增长”活动，协调解决企业市场拓展、融资、用地等困难，工业经济实现企稳回升，规上工业总产值实现1330.4亿元、增加值增长10.2%。大力实施大企业大集团培育计划，重点扶持小微企业发展，新注册小微企业1367户，新增规模以上企业71户。加快推进产业园区建设，新拓展面积56.6平方公里，园区基础设施和功能进一步配套完善。切实发挥生产要素对稳增长的保障作用。搭建合作平台，加强银企对接，支持实体经济发展，新增贷款90.29亿元、增速居全省第6位；加快推进城乡建设用地增减挂钩和工矿废弃地复垦利用试点，获批新增建设用地指标1033公顷。继续实施电煤调运“绿色通道”制度，协调争取天然气指标和直购电政策，广安发电厂、玖源化工生产用煤用气得到保障，诚信化工、华油LNG等重点企业享受直购电政策。

着力推动经济转型升级发展。坚持在增量中调结构，在发展中促升级。大力实施创新驱动发展战略。加快科技创新平台建设，深化与中关村、中国农科院务实合作；发展“院士经济”，与尤政院士团队建设清华大学广安新能源研究院，与李长明院士团队设立西南高端产业技术研究院；实施重大科技成果转化项目15项、技术创新项目37项，成功创建省级企业技术中心5家，培育国家级高新技术企业9家，获得发明专利授权15件，荣获省政府科技进步奖4项，高新技术产业总产值达399.9亿元、增长33%。坚持走新型工业化发展路子。继续改造提升传统产业，实施重大技改项目78个，完成技改投入158.3亿元；关闭地方小煤矿8处、淘汰落后产能65万吨；出台支持精细化工、生物医药、电子信息、节能环保新材料、新型住宅制造、高端装备制造等六大高端成长型产业及百亿产业发展的政策措施，潜力产业和战略性新兴产业实现产值249亿元，占比提高2个百分点。节能降耗成效明显，预计单位工业增加值能耗下降3%，单位GDP能耗下降2%。推动现代服务业改革发展。扎实推进省级服务业综合改革试点市和现代物流业发展试点示范市建设，制定实施促进服务业发展的意见，重点培育发展电子商务、现代物流、现代金融、科技服务、养老健康、通信服务等六大新兴先导型服务业，实现增加值79.6亿元，占服务业比重达28.3%；旅游业快速发展，新创建国家4A级景区4个，成功举办“5·19”中国旅游日广安主会场活动，红色文化影视城一期投入营运，全市实现旅游收入194.6亿元、增长43.8%；保险业健康发展，理赔金额11.7亿元。全年实现服务业增加值281亿元、增长11.7%。积极发展现代农业。加快推进国家现代农业示范区建设，大力实施现代农业和产村相融“111”工程，建成万亩以上现代农业园区83个87万亩、畜禽标准化规模养殖场（小区）5227个；狠抓农产品品牌建设，新发展无公害、绿色、有机农产品和地理标志农产品47个，成功推出“邓小平故里华蓥山优质农产品”公用品牌；粮食产量达到185.7万吨，农业实现增产增效。三次产业结构持续优化，二、三产业比重分别提升0.1、0.9个百分点。经济发展效益向好，地方一般公共预算收入完成46亿元、增长17.4%，税收占比提高到63.6%。

统筹推进城乡发展。加强规划引领，创新管理机制，突出以人为核心的城镇化建设，全市新型城镇化沿着正确的方向有序推进。大力推进城乡基础建设。加快东南片区、官盛新区建设，推进化龙沟、岔路口等片区旧城改造，完成主城区金安大道、建安路、五福西路市政配套建设，开工建设保障性住房和棚户区改造38376套，新增城镇面积21.5平方公里，城镇化率提高1.52个百分点。推进智慧城市和“宽带乡村”工程建设，建成投运城市公共自行车服务系统，全面启动“双违”治理，城市管理水平不断提高。启动建设30个特色集镇，13个镇获评全国重点镇，建成幸福美丽新村30个、新农村聚居点488个；8个新型房屋示范点564套房屋全部竣工。抓好农田水利项目建设，全民水库增容改造、西溪河堤防一二期工程全面完工，七一水库扩改建工程、关门石和天池湖水库渠系配套项目加快建设，猫儿沟、回龙寺、应家沟水库开工建设，新增恢复改善有效灌面18.7万亩，新建供水工程663处，解决19.17万人饮水问题。农业机械化综合水平达到55.2%。着力完善城乡交通体系。彭枣路、流杯滩渠江大桥、广岳大道等工程建成通车，广安渠江航道、广华大道等工程加快建设，渠江官盛大桥开工建设，广安市过境高速公路东环线及渝广高速支线、广安枣山客运枢纽站等项目前期工作快速推进；建成农村公路1221.8公里，完成公路安保设施600公里；新发展农村客运片区22个，新增和更新农村客运车辆175辆。综合治理城乡生态环境。启动实施华蓥山区生态修复工程，大熊猫入住华蓥山野化放归培训基地。全面开展“洁净水”行动，重点推进西溪河、芦溪河、大洪湖、五排水水库及长滩寺河等流域污染治理，建成乡镇生活污水处理厂（站）96座，嘉陵江、渠江出境断面水质达标，城区、乡镇集中式饮用水源地水质达标率分别为100%、85%。加强大气污染防治，PM2.5空气自动监测站、邻水县机动车尾气检测中心建成投运。市垃圾焚烧发电厂基本竣工。巩固国家森林城市创建成果，加强石漠化、地质灾害、水土流失治理，森林覆盖率达到38.9%。

持续深化改革扩大开放。抓紧启动重点领域改革。按照市委四届七次全会决定的改革“时间表”和“路线图”，出台实施了一系列重大改革方案，启动或完成32项改革事项。在简政放权上，市本级取消、下放40项行政审批事项；实施商事制度改革，新登记企业增长58.9%。在政府机构改革上，农业与农机畜牧、卫生与人口计生、教育与体育、科学技术和知识产权、财政与国资等职能整合全面完成，落实工商、质监下划地方管理，事业单位分类改革稳步推进，广电网络完成改制。在财税体制改革上，开展税收分享体制改革，完善市区财政体制，推进“营改增”改革试点，深化绩效预算管理和国库管理改革，启动综合治税平台建设。在投融资体制和金融改革上，推出鼓励社会投资重大项目，扩大民间投资领域；探索实施PPP模式治理水环境；推进企业债券发行，邓小平故里发展基金启动运营；武胜信用联社改制成农村商业银行，华蓥和邻水信用联社改制正式启动。在农村综合配套改革上，全面开展农村集体土地承包经营权确权登记颁证工作，新增土地流转面积13.14万亩；大力培育新型农业经营主体，新增家庭农场9个、农民专业合作社295个。务实推进对外开放合作。加快推进川渝合作示范区、承接产业转移示范区建设，深化与长三角、珠三角等发达地区的合作，与重庆经济协作区、川东北经济区在基础设施、产业发展、公共服务等领域合作取得新的进展；依托“中外知名企业四川行”、西博会、渝洽会等重大平台，有效开展“感恩小平·投资广安”系列投资促进活动，广安-深圳合作产业园挂牌并启动建设，成都农商行、重庆银行入驻广安，全年引进项目950个、到位资金815.6亿元。调整外贸出口扶持政策，加强生产型出口企业培育，鼓励企业开拓国际市场，实现外贸出口10.7亿美元、增长9.6%，总量居全省第4位。

大力推进多点多极支撑发展。制定出台支持县域经济改革发展政策措施，切实为区市县发展松绑减负，为园区建设破除体制机制障碍，县域经济呈现出蓬勃发展的良好势头。广安区建成官盛新区10平方公里雏形，启动临港都市产业园建设，进入四川省首批旅游十强县。前锋区基本形成15平方公里城市骨架路网，城市综合体规划设计方案获得国家级金奖，轻纺和装备制造两大主导产业快速壮大。华蓥市资源枯竭型城市加快转型，电子信息产业实现由零部件加工到手机整机生产的历史跨越，高兴铁路物流园区纳入省级规划。岳池县被评为全省县域经济发展先进县，大力发展医药产业集群，入园企业快速增加。武胜县创建为全国休闲农业与乡村旅游示范县，宝箴塞旅游区、白坪-飞龙乡村旅游度假区创建为国家4A级景区，太极湖列为国家级水利风景区。邻水县成功创建为全省生产性服务业功能示范区，渝广共建机电产业园快速发展，以汽摩配件为主的机电产业成为首个百亿级产业集群，高滩工业园被授予“重庆空港工业配套园”。广安经开区加快建设千亿级产业园区，精细化工、新材料、新型住宅等产业链条不断延伸，拓展园区面积10.5平方公里，基本形成30.8平方公里道路骨架。枣山园区10平方公里基础设施基本完成，一批商贸物流专业市场快速建设。协兴园区建成20平方公里骨干路网，生态、文化、旅游等产业龙头项目相继落地，爱国主义教育基地影响力进一步提升。同时，充分运用市场法则，推动投融资平台建设，广安发展建设集团全年直接融资5.66亿元，广安交通投资建设开发集团、广安金财投融资集团完成组建并投入运行。

全面加强民生工作和社会建设。始终把改善民生摆在突出位置。整合公共资源，加大民生投入，民生支出131.1亿元，占一般公共预算支出的70%。完成省市民生项目143个，解决了一批群众关心的上学难、就医难、出行难、饮水难等实际问题。全面实施精准扶贫，减少贫困人口9.25万。积极应对＂9o13＂特大暴雨洪灾，灾后重建有力有序开展。城镇居民人均可支配收入24475元、增长10.2%，农民人均纯收入9514元、增长12%。切实加强就业促进和社会保障工作。积极开发公益性就业岗位，新增城镇就业5.01万人，城镇登记失业率控制在3.58%；建立统一的城乡居民养老保险制度，稳步推进城镇居民基本医疗保险、企业职工基本养老保险市级统筹，城乡居民大病保险和灵活就业人员参加职工基本医疗保险制度全面实施，社会保险覆盖人数达到287.16万人。连续十年上调企业职工养老保险待遇，两次提高城乡低保标准。社会养老服务体系加快建设。统筹推进社会事业健康发展。部省共建教育改革试验区建设取得新进展。启动第二期学前教育三年行动计划，新建城乡公办幼儿园21所；大力实施义务教育学校标准化建设，新改扩建学校106所，城镇学校大班额问题得到有效缓解；实施特殊教育提升计划，适龄残疾儿童入学率达92%；广安职业技术学院创省级示范性高职院校接受中期验收，武胜职专创国家中职改革发展示范校通过阶段性评估；与北京师范大学合作建设邓小平城乡发展学院。全民健身运动和群众体育活动蓬勃开展；竞技体育成果丰硕，在省十二运会上取得16枚金牌、创历史最好成绩，在省残运会及特奥会上名列金牌榜第三名。积极创建全国文明城市，城市公共文明指数位居全省地级城市第一名、全国地级城市第三名，目前广安作为中央文明委拟命名的第四届全国文明城市，已完成公示。加快构建现代公共文化服务体系，建成广安大剧院、市文化中心，文化创意、文艺演展、城市影院等文化产业加速培育。文化遗产保护得到加强，武胜县被命名为“中国民间文化艺术之乡”，岳池曲剧列入省级非物质文化遗产名录。县级公立医院综合改革深入实施。初步建立分级诊疗制度。新型农村合作医疗参合率达99.2%。＂120＂急救指挥系统实现全市统一调度，市全科医生培训基地建成投运，新增民营医疗机构6家，传染病发病率低于全省平均水平；“单独两孩”政策得到落实，人口自然增长率2.36‰。全面推进社会依法治理。广泛开展法治示范创建活动，“六五”普法获全国表彰，“一标三实”基础信息采集综合绩效全省第一；加强食品药品和工商行政监管，清理整顿投融资秩序；狠抓煤矿、非煤矿山安全生产，集中开展道路交通安全综合整治攻坚年行动和火灾防控专项行动，全市安全生产事故起数、死亡人数分别下降24.3%、10.4%，未发生重特大安全生产事故；深化平安广安建设，成立广安市国家安全局，建立应急联动指挥中心，有效组织重大活动安全保卫，全面开展“五大民生警务”活动，积极预防和化解社会矛盾，依法打击各类刑事犯罪活动，有效防控暴力恐怖犯罪，社会大局保持稳定。

加强政府自身建设和管理创新。加快转变政府职能，依法规范权力运行，持续深化正风肃纪，政府公信力和执行力显著增强。认真开展群众路线教育实践活动。全面贯彻落实中央八项规定、省委省政府十项规定，强力整治“四风”突出问题，扎实开展＂9+3+X＂专项整治，重点纠正“说假话、执行不力、作风不实”等问题，查处“四风”问题案件180件，党纪政纪处分218人，全市政风行风明显好转。大力推进法治政府建设。深入实施依法治市纲要，坚持市政府常务会学法，完善市政府法律顾问团制度，强化行政决策合法性审查，规范行政执法裁量权，加强行政决策风险评估，推进行政权力依法规范公开运行。深化政务信息公开，大力推行“网络理政”、“在线访谈”，有效促进了政民交流互动。主动接受市人大及其常委会的法律监督、工作监督和市政协民主监督，充分听取各民主党派、工商联、无党派人士和人民团体的意见，办理人大代表议案建议154件、政协提案248件，办复率和满意率均为100%。切实加强廉政风险防控。出台规范政府投资项目招投标、土地及矿产资源出让、公共资源交易、财政资金管理等规范性文件21个，努力把行政权力关进制度的笼子；强化财政、审计监督和行政监察，主动接受群众监督和舆论监督，强化了在监督下工作的机制和氛围。

国防建设、民兵预备役、双拥共建和人民防空工作得到加强，统计服务、国资管理、粮食储备、食盐储备、防震减灾、住房公积金管理、信息化建设、供销、气象、外事、侨务、港澳台事务、民族宗教、档案、保密、地方志、老龄事业、邮政、烟草专卖等工作取得新成绩，石油、电力、供水、通信等保障工作取得新进展，工会、共青团、妇联、科协、残联、贸促会、社科联、文联、红十字会、关工委以及各民主党派、工商联等做出新贡献。

各位代表，过去一年，我们成功筹办了邓小平同志诞辰110周年纪念活动，实现了中央和省委满意、小平同志家人满意、人民群众满意的目标。我们抢抓纪念活动新机遇，广泛开展感恩作贡献活动，凝聚了发展合力；主动适应经济新常态，下功夫推动产业转型升级，提升了发展质量；探索改革开放新举措，着力破除体制机制障碍，增强了发展动力；着力办好民生大事实事，切实增进人民福祉，顺应了群众新期盼。

各位代表，过去一年，面对复杂严峻的宏观经济形势，全市广大干部群众始终保持“逢山开路、遇水架桥、风雨无阻、昼夜兼程”的精神状态，满怀激情干事创业，凝心聚力攻坚破难，跨越发展的成绩来之不易，感恩奋进的历程弥足珍贵。在此，我代表市人民政府，向全市干部群众，向人大代表、政协委员，向各民主党派、工商联、无党派人士、人民团体和各界人士，向驻广部队、武警官兵和公安民警，向所有支持广安改革发展的同志们、朋友们，表示衷心的感谢！

各位代表，总结去年工作，我们也清醒地看到，广安发展中还面临不少困难和问题，主要表现在：经济下行压力依然较大，一些行业企业短时期内难以走出困境；县域经济实力不强，新的经济增长点尚需加速培育；结构调整、转型发展任务还很艰巨；地方债务、金融等领域潜在风险逐步显现，深化改革、去产能化过程中的社会压力增大；基本公共服务均等化还需加力加速，城乡群众生产生活中的一些突出问题还有待解决；政府自身建设方面，在工作落实上还有一定差距，“庸懒散浮拖”等不良现象还不同程度存在，依法行政和反腐倡廉建设还需加强。这些问题和困难，我们将高度重视，认真加以解决。

2015年工作意见

2015年，是实施“十二五”规划的收官之年，也是改革发展任务繁重的一年。确定今年政府工作思路，制定经济社会发展目标，全面推动各项工作，需要我们科学研判、充分权衡几个方面的因素。一是准确把握经济新常态的特征和要求。经济新常态是中央对我国经济发展阶段性变化作出的重大战略判断，其基本特征是“速度变化、结构优化、动力转换”；省委立足四川实际，鲜明提出“五个方向性要求”，并强调在新常态下，绝不意味着可以放松发展，更需要加大力度，保持一个高于全国的合理增长，这为我们正确认识形势、与时俱进做好经济工作，提供了基本方略和重要遵循。面对经济新常态，我们既要主动适应发展速度从高速向中高速的转变，处理好稳增长和调结构的平衡关系，稳住发展速度，又要着力转方式调结构，推动经济从要素驱动、投资驱动向创新驱动转型，在转型发展中形成增长新动力，努力提高经济发展质量和效益。二是全面审视我市的基本市情和现状。对广安来说，“人口多、底子薄、欠发达”的基本市情还没有改变，“发展不够、发展水平不高”的主要矛盾依然突出。要与全国全省同步全面建成小康社会，在全省多点多极中实现次级突破、奋勇争先，时间更加紧迫、任务更加繁重。这就需要我们更加积极主动作为，跳起摸高、加压奋进，坚定不移抓好发展第一要务，按照市委快节奏、高速度、超常规发展的要求，推动经济持续快速发展，保持高于全国全省的增长速度，朝着“两个跨越”目标奋力迈进。三是充分认识广安发展的机遇和潜力。当前，广安正处于跨越发展、大有作为的关键时期，全面深化改革将释放更多的制度红利，加快转型发展将培育新的产业优势，新型城镇化、多点多极支撑发展、川渝合作示范区、承接产业转移示范区建设正成为广安追赶跨越的强大引擎，国家推进“一带一路”和长江经济带建设又给广安带来新的重大机遇。只要我们充分挖掘发展潜力，用好多重发展机遇，努力在薄弱环节、比较优势、中省支持的重点中寻找新的增长点，就会形成保持经济快速增长的内生动力，推动广安经济发展换挡不失速、调速不减势、量增质更优。

基于上述考虑，今年政府工作的总体要求是：深入贯彻党的十八大、十八届三中、四中全会和省委十届四次、五次全会精神，在贯彻落实中央、省委、市委重大决策部署上凝神聚焦发力，按照市委四届八次全会部署，主动适应经济发展新常态，坚持稳中求进工作总基调，以提高经济发展质量和效益为中心，深入实施“三大发展战略”，加快转型发展，狠抓改革攻坚，推进依法治市，加强民生保障，强化风险防控，促进全市经济健康较快发展和社会和谐稳定。主要预期目标是：全市地区生产总值增长9%以上，全社会固定资产投资增长15%以上，社会消费品零售总额增长12%以上，地方一般公共预算收入增长10%以上，城镇居民人均可支配收入增长10%以上，农民人均纯收入增长11%以上，居民消费价格涨幅控制在3%以内，人口自然增长率控制在5.4‰以内，完成省上下达的节能减排任务。实现上述目标，我市的地区生产总值和固定资产投资将双双突破1000亿元，广安的经济发展将上到一个新的台阶。

按照上述总体要求和预期目标，今年重点抓好以下几方面工作：

一、突出投资消费拉动，努力保持经济较快增长

充分发挥投资消费拉动经济增长的主引擎作用，从需求方面施策，从供给方面发力，努力扩大投资规模和消费需求。

保持投资持续增长。准确把握国家产业政策、中省投资方向，开展具有全局性、战略性的重大问题研究，科学编制“十三五”规划。用好川渝合作示范区、承接产业转移示范区等支持政策，主动对接生态环保、重大水利、交通、能源等7大类建设重点，精心策划包装、筛选上报一批重大项目，确保更多重大项目挤进中省“十三五”规划盘子。稳定和扩大政府公共投资规模，运用政府与社会资本合作的多种投融资模式，加强生态环保、重大水利、交通、能源等领域的项目建设，增加公共产品有效供给。充分挖掘产业投资潜力，提高研发创新和高端制造业投资比重，支持企业加大技术改造和创新投入，促进产业投资稳定增长。加快实施517个投资1000万元以上的重大项目（含市列重点项目），确保重大项目投资完成682.5亿元。

努力扩大消费需求。顺应群众个性化、多样性的消费需求，重点挖掘养老健康家政、信息、旅游、住房、教育文化体育等领域消费潜力，培育新的消费热点。积极参加“惠民购物全川行动”“川货全国行”活动，大力举办各类展会展销活动，提高名优特新产品市场占有率。支持城乡消费设施和信息基础设施建设，继续打造主城区中央商务区和县城核心商圈，加快武胜、邻水农产品交易市场建设，升级改造农贸市场，推进家居建材、汽车销售等专业市场建设，完善农村电商配送网络，着力引进电商企业在广安设立总部或区域结算中心，加快构筑城乡现代流通体系。认真落实增加城乡居民收入各项政策，努力提高中低收入群众收入，不断增强群众消费能力。

积极发展外向型经济。积极拓展市场空间，提高出口创汇对广安经济的贡献率。优化外贸出口结构，支持生产型出口企业集聚发展，重点打造华蓥电子信息产业、前锋轻工产业、邻水装备制造基地。继续争取广安设立海关和商检机构，积极服务外贸企业发展。深入推进“万企出国门”活动，支持一批成长型、潜力型企业拓展外贸业务。鼓励优势企业“走出去”，开展工程承包和劳务合作。提高外资利用水平，实际利用外资4000万美元。

二、坚定走转型发展之路，积极发现培育新的增长点

坚持创新发展、高端发展，依托科技进步和全面创新，推动产业结构优化升级，不断提升经济发展质量和效益。

努力促进工业提质增效。依托创新培育工业发展新优势，大力发展六大高端及百亿产业，推进产业集群集约发展，实现产值345亿元，占比提升3.2个百分点。整合政府技改支持资金，抓好重点企业技术改造，完成技改投资182亿元、增长15%。深入实施大企业大集团培育计划，做大做强一批龙头企业，培育发展中小微企业，新增规上企业50户、小微企业1200户。持续优化工业布局，抓好重点产业园区建设，推动产业园区提档升级、转型发展。继续发挥市场作用化解过剩产能，淘汰落后产能。

加快发展现代服务业。经济转型发展，突破口在服务业。坚持把服务业发展放在更加重要位置来抓，扎实推进服务业综合改革试点市和现代物流业试点示范市建设，推动服务业发展提速、比重提升、水平提高，实现服务业增加值315亿元，增长10%。优先发展六大新兴先导型服务业，力争在服务业中占比提升0.5个百分点。加快重点旅游项目建设，打造精品旅游景区景点，配套完善旅游基础设施，加强省内外旅游交流合作，整体提升旅游业发展质量，力争旅游收入增长20%以上。推进6个省列“双百工程”项目建设，努力建设区域服务中心城市。实施“主辅分离”工程，加快发展与工农业配套的电子商务、物流配送、文化创意、科技服务等服务业，进一步提高生产性服务业在服务业中的比重。

着力推进科技创新驱动。对接产业链布局重大关键技术攻关、科技成果转化与创新产品培育，强化技术研发与推广运用。加快完善创新平台，创建省级技术创新工程示范县3个。支持企业提升自主创新能力，新培育国家级高新技术企业5家、省级创新型企业5家。鼓励和引导各类创业投资基金重点扶持小微企业创新，推进科技企业孵化器建设。深入实施人才强市战略，进一步激励科技人才创新创业。深入推进国家商标战略实施示范城市建设。做好创建国家知识产权强县工程试点工作。

三、加快转变农业发展方式，促进农村发展农民增收

始终把解决“三农”问题作为工作的重中之重，围绕农民增收这个核心，大力推进农业现代化和农村改革发展。

加快发展现代农业。坚持走产出高效、产品安全、资源节约、环境友好的现代农业发展道路，以国家现代农业示范区建设为抓手，加快推进产村相融＂111＂工程建设，新建现代农业产业基地16万亩，确保粮食稳产、农业增效。大力推进农业产业化经营，深入实施品牌发展战略，加大力度推介营销“邓小平故里华蓥山优质农产品”公用品牌，不断提升广安农产品影响力和市场竞争力。强化农业科技创新和重大技术推广，提升农业机械化水平，加强高素质职业农民队伍培育。加快建设猫儿沟、回龙寺、应家沟水库和渠江堤防工程，开工建设向阳桥水库，搞好灌区节水改造，进一步改善现代农业基础条件。

全面深化农村改革。积极争取改革试点试验。完成农村集体土地承包经营权确权登记颁证，同步推进“多权同确”。引导土地经营权有序流转，发展各类新型农业经营主体。建立完善农村产权评估、交易机构，促进农村产权流转交易市场健康发展。加强涉农资金统筹整合和管理，推进财政支农资金股权量化改革和农村土地流转收益保证贷款试点，探索建立农村资金互助合作组织。

持续推进新农村建设。坚持“物的新农村”和“人的新农村”齐头并进，统筹推进基础设施、新型村庄、主导产业、公共服务、社会管理和生态文明建设，建成幸福美丽新村220个，建设新民居2.3万户、农村廉租房4000套。加强农村传统文化、乡土文化保护工作。建立农村留守老人、留守儿童、留守妇女关爱服务体系，进一步优化农村养老、教育、医疗等公共服务，让“三留守”有依靠有保障。

多渠道促进农民增收。稳定农产品价格，持续增加家庭经营收入。加大各类农民技能培训资源整合，稳定农民转移输出规模，做响劳务品牌，提升劳务经济质效。大力发展农产品加工、运输物流、休闲农业、乡村旅游等二三产业，拓展非农就业增收空间。发展壮大村集体经济，开展农村集体资产股份制改革试点。认真落实强农惠农政策，切实增加农民转移性收入。

四、全面深化改革开放，切实增强发展动力活力

坚持以政府自我革命牵引重点领域改革，推动高水平开放合作，不断激发经济发展新动力、新活力。

进一步深化重点领域改革。深入推进简政放权、放管结合的改革，清理优化行政权力目录，全面取消非行政许可审批事项，实现省市县联网审批。深入推进商事制度改革，提高市场准入效率。深化投融资体制改革，实施新版政府核准投资项目目录，规范政府投资项目和融资平台管理；推进公共资源交易流程化机制改革。深化财税体制改革，完善政府预算体系和控制方式，规范地方政府债务管理；建立涉税三方信息采集交换平台和分析应用机制；落实清理规范税费优惠政策，继续开展“营改增”等税制改革。推进金融改革创新，支持县级信用联社改制组建农村商业银行，加快发展服务小微企业的中小微金融机构，加强多层次资本市场体系建设，改革发展现代保险业，建立和完善金融风险防范机制。积极稳妥推进价格改革，最大限度缩小政府定价范围。大力推行政府购买服务，有序推进公务用车制度改革。深化事业单位分类改革，探索规范公益类事业单位管理。结合养老保险并轨改革完善机关事业单位工资制度。深化国有企业改革，发展混合所有制经济，探索国有资产保值增值新路径。

进一步推进区域合作发展。围绕川渝合作示范区建设“2015年初见成效”的目标，用好用活川渝合作示范区优惠政策，筛选实施一批渝广年度合作项目，加快渝广两地交通互联、产业相融、生态共建、服务共享。加快推进承接产业转移示范区建设，主动对接国家南向西向开放和“一带一路”倡议，深化与长三角、珠三角等发达地区的经贸合作；推进广安-深圳合作产业园建设，力争在机制创新和项目落地上有新的突破。大力实施川东北经济区发展规划，加快推进区域重点合作项目，力争在旅游、交通、物流等领域合作取得新进展。加强与友好城市的交流，深化经贸、文化、科技、教育等领域的合作，努力提升开放合作水平。

进一步加强重点产业招商。围绕六大高端及百亿产业、六大新兴先导型服务业及现代农业产业，精心谋划和组织开展重点区域投资促进活动，积极参加“中外知名企业四川行”、渝洽会、西博会等展会活动，继续开展“感恩小平·投资广安”“中外知名企业广安行”等专题招商活动，重点对接高端产业和产业高端，实现产业招商大的突破。力争签约引进项目900个、形成固定资产480亿元。抓好已签约项目跟踪落地工作，努力提高招商引资项目履约率和资金到位率。

五、强化城乡统筹和多点多极支撑，不断优化经济空间格局

坚持规划引领，优化空间布局，加速培育新的经济增长点，继续保持多点多极竞相发展的良好势头。

加速推进新型城镇化。积极探索“多规合一”规划编制，突出分区规划和控详规编制。加快主城区与岳池、华蓥、前锋城市组团融合发展，推进武胜、邻水区域中心建设，加快建设50个特色集镇，新增城镇面积18平方公里，城镇化率提高2个百分点。深入推进城镇化改革，落实户籍制度改革方案，推进农村转移人口均等享受城镇基本公共服务。集中开展“双违”治理，严格规范用地和建设秩序。创新机制和办法破解城镇化发展难题，鼓励社会资本参与新型城镇化建设；深化土地要素配置和差别化用地机制改革，支持城镇低效用地再开发，拓展城乡建设用地增减挂钩、工矿废弃地复垦等试点，不断满足新型城镇化建设用地需求。

持续发展壮大县域经济。深入贯彻落实加快县域经济改革发展政策措施，促进县域经济追赶跨越、竞相发展。支持广安区现代服务业、前锋轻纺加工、华蓥电子信息、岳池医药、武胜节能环保、邻水装备制造等主导产业做大做强，努力壮大县域经济产业支撑。充分发挥民营经济在推动县域经济发展中的主体作用，有效激发民营经济发展活力和创造力，不断壮大县域经济实力。

做实做强市级园区和国有企业。围绕建设千亿园区目标，推进广安经开区奎阁、新桥园区建设，着力做强精细化工、新材料两大主导产业，做实有色金属、装备制造、新型住宅三大优势产业，力争建成全省规模最大的精细化工基地和全省的新材料基地。围绕打造对接重庆、辐射川东北商贸物流集散地，加速推进枣山园区物流通道和配套设施建设，积极引进大中型商贸综合体、专业市场、现代物流企业，指导企业创新经营机制，科学配置业态，搞活经营，加快构建枣山总部经济集群。围绕建设世界级旅游目的地，加快完善协兴园区基础设施配套，着力打造集爱国教育、教育培训、文化创意、休闲度假等于一体的文化旅游产业新城；加强华蓥山旅游资源保护和地质灾害区治理，提档升级石林、天意谷景区，加快建设华蓥山自然保护区和宝鼎禅修文化旅游景区，积极创建华蓥山国家级旅游度假区，推动旅游资源优势向旅游经济优势转变。支持市发展建设集团、交通投资集团、金财集团抓好主营业务，搞好经营管理，实现国有资产保值增值，在市域经济发展中发挥更大的作用。

强化城乡基础设施建设。科学编制城镇地下管线综合规划，试点推进城镇地下综合管廊建设，加快建设和改善排涝防洪、垃圾污水处理等公共基础设施，不断增强城镇承载能力。加速构建城乡互联互通交通体系，开展广安（高兴）-重庆（黔江）铁路建设前期工作，全面竣工兰渝铁路武胜段，加快推进港前大道、广华大道等重大交通基础设施建设，启动实施广安市过境高速东环线及渝广高速支线、罗渡大桥等工程，开工建设省道203广安龙台（达州界）至兴平（岳池界）公路，力争遂广高速公路蓬溪界至岳池齐福段、国道350线邻水石滓至重庆界完工，富流滩二线船闸、渠江航道整治全面竣工；提升改造县乡公路200公里。推进重点输变电工程建设，加快实施农村电网升级改造，解决农村地区电压过低、电力供应不足问题。实施智慧城市发展战略，加快4G网络建设、三网融合、光纤入户步伐，加快实施“宽带乡村”、“信息惠民”等工程，不断提升城乡信息化、数字化水平。

六、全面发展民生社会事业，努力提高群众幸福指数

更加注重保障基本民生，更加关注低收入群众生活，更加重视基本公共服务均等化，务实抓好新形势下的民生工作。

扎实推进民生工程。建立民生工程投入稳步增长机制，确保一般公共预算民生支出比重保持在65%以上。全面完成省定“十项民生工程”和20件民生大事。集中财力办好市定民生实事，完成通村公路建设600公里、异地扶贫搬迁1000户、整治山坪塘600口、新改扩建农村敬老院20所，以及资助贫困中小学生、升级改造农贸（菜）市场、新建乡镇妇女儿童之家、新增城区停车位等8大类25项惠民项目。

全面加强社会保障。进一步完善落实就业促进、创业引领、职业培训和服务均等化政策措施，抓好高校毕业生为重点的青年群体就业，做好农村转移劳动力、城镇就业困难人员、随军家属、退役军人就业工作，促进化解过剩产能失业人员再就业，实现城镇新增就业3.4万人，城镇登记失业率控制在4%以内。逐步建立覆盖城乡的社会保障体系，巩固和完善工伤、生育、失业保险市级统筹办法，全面实施城镇居民大病保险、企业职工基本养老保险市级统筹和城镇居民基本医疗保险统收统支制度。提高新农合保障水平，完善最低工资、低保标准增长机制，加强医疗救助和临时救助制度建设。加快异地就医结算平台建设，实现省内异地就医同步结算。关注和保障弱势群体权益。

协调发展社会事业。优先均衡发展教育事业。基本完成部省共建广安市教育改革发展试验区建设任务。继续实施第二期学前教育三年行动计划。全面改善义务教育学校办学条件，推进义务教育均衡发展。加大省级示范性普通高中建设支持力度，积极探索举办综合高中。大力构建现代职业教育体系，支持广安职业技术学院建成省级示范性高职院校。关心支持特殊教育发展。推进与西南大学、四川理工学院合作办学，加快邓小平城乡发展学院建设。鼓励支持民办教育健康发展，提升民办教育办学水平。切实加强学校德育、艺体、健康教育，深入实施素质教育。扎实抓好师德师风建设，提升教师队伍整体素质。积极推动文化繁荣发展。大力实施城乡“结对子·种文化”工程，促进城乡基本公共服务均等化；扎实推进文化惠民工程，加快建设市博物馆、县级文化场馆，推进公益文化设施免费开放常态化。加强优秀传统文化的传承弘扬和重点文物保护，抓好全市第一次全国可移动文物普查工作。推动文艺创作繁荣发展，丰富文化产品供给。培育壮大文化产业，加强文化市场监管，净化社会文化环境。继续开展群众性精神文明创建活动，巩固提升创建全国文明城市成果。启动县级体育场馆和全民健身中心建设，加强社区群众体育设施建设，促进群众体育、竞技体育和体育产业发展。扎实抓好卫生计生服务。加快市人民医院第二住院大楼、市妇幼保健院和前锋区人民医院建设，启动岳池县和邻水县中医院、华蓥广能医院迁建工程。完善重大疾病防控体系和突发公共卫生事件应急机制，开展“平安医院”创建，提升重大突发公共卫生事件应急处置水平。鼓励和引导社会资本举办医疗机构和康复疗养机构，全力推行分级诊疗制度和试点医师多点执业。继续实施“单独二孩”政策，提高计划生育服务管理水平。做好国有档案滚动移交进馆工作。促进慈善事业健康发展。

深入开展扶贫攻坚。认真落实责任、权力、资金、任务“四到县”制度，坚持区域扶贫与精准扶贫“双轮驱动”，动员社会力量参与扶贫，深入推进“五大扶贫工程”，减少贫困人口5.3万以上。做好新建水库移民安置工作，完成移民后扶整村推进示范片建设任务。抓好“9·13”灾后重建工作。

七、加强生态环境保护，推动绿色低碳循环发展

顺应人民群众对清新空气、洁净水质和优美环境的殷切期待，加快建设资源节约型、环境友好型社会，努力让广安天更蓝、地更绿、水更净。

加强污染综合治理。坚持源头严防、过程严管、后果严惩、标本兼治，铁拳治理大气、水、土壤、重金属等重点污染，专项治理燃煤电厂、水泥企业、煤矿企业等工业污染，建筑工地施工、道路运输等扬尘污染，加强机动车尾气污染监测整治。扎实开展“洁净水”八大行动，不断改善水系、水面、水质、水岸环境质量。推进嘉陵江生态经济带建设，加强渠江广安段水污染治理与饮用水源保护。广泛开展农村面源污染、生活垃圾专项治理行动，加快推进区市县污水处理厂和城镇污水垃圾处理设施建设，加强运行监管，提高运行效率，着力消除城乡“黑臭河”。严格环境准入，优化环评审批，建立环境污染公共监测预警机制。狠抓污染企业挂牌整治，加强环境风险隐患排查，依法打击环境违法犯罪行为。开展企业环境信用评价，建立环保“黑名单”制度。

推进集约节约发展。积极创建全国循环经济示范城市，加快推进广安经开区国家级循环经济示范试点建设，强化结构减排、工程减排和管理减排，推广应用节能减排装备和技术。落实最严格水资源管理制度，严控用水总量，提高用水效率，建设节水型社会。严格土地用途管制，加强矿产资源保护与合理开发管理，提高土地投资强度和产出效益。落实节能减排目标责任制，力争单位工业增加值能耗下降3.5%以上、单位工业增加值用水量下降7.5%、工业固废综合利用率提高到75%，二氧化硫、化学需氧量、氨氮和氮氧化物排放量控制在目标范围内。

强化生态保护与建设。开展生态保护红线划定工作，保证和拓展生态建设用地。加强自然生态保护修复，大力实施天然林保护、石漠化治理等重点工程，启动实施新一轮退耕还林工程，强化生态功能区、湿地公园建设和管理。抓好生物多样性保护，加强大熊猫野化培训工作。推进国家级生态文明市、生态文明县（区市）和省级生态工业园区建设。完善自然资源有偿使用、用途管制、生态补偿等制度机制，稳步推进生态文明体制改革。

八、深入推进依法治理，切实维护社会和谐稳定

坚持以法治为引领，完善社会治理机制，加强公共安全体系建设，努力营造安居乐业的社会环境。

创新社会治理方式。深化全民“六五”普法教育，扎实开展法治示范创建活动，加快建设覆盖城乡的公共法律服务体系。坚持用法治思维和法治方式解决社会矛盾，解决好土地征用、房屋拆迁、环境治理等引发的矛盾纠纷。认真开展重大决策社会稳定风险评估，深化创新“大调解”工作机制，依法规范信访秩序。深化城乡社区治理体制创新，全面推进网格化服务管理。推进社会组织监管改革，引导社会组织健康发展。推动政府治理和社会自我调节、行业自律、居民自治良性互动，支持工会、共青团、妇联、工商联等人民团体参与社会治理，形成多元共治局面。

扎实抓好安全生产。健全完善安全监管责任体系，突出抓好重点领域隐患排查和专项整治，抓好矿山、非煤矿山、水陆交通、民爆危化品、建筑施工、森林防火和公共聚集场所等安全工作，深化巩固道路交通综合整治和火灾防控成果，有效遏制重特大安全事故发生，确保安全生产形势持续稳定好转。进一步加强食品药品安全监管，加快建立食品可追溯制度和质量标识制度，严厉打击食品药品安全违法行为，确保人民群众“舌尖上的安全”。

深化平安广安建设。加强社会治安综合治理，深入开展“反邪教”专项斗争，强化反恐防暴工作，依法打击各类违法犯罪；纵深推进“五大民生警务”活动，巩固“一标三实”基础信息采集成果，创新流动人口和特殊人群服务管理，推动社会治安防控法治化、社会化。加强社会信用体系建设，实施信用“黑名单”制度；持续整顿和规范市场秩序，进一步营造公平竞争、诚实守信的市场环境。清理整顿投融资中介机构，化解和防范民间金融风险。完善防灾减灾和应急救援体系，继续抓好地质灾害防治工作，有效防范和应对重大自然灾害。认真落实民族宗教政策，加强国防动员和后备力量建设，做好人民防空、国家安全和保密工作，努力维护社会大局稳定。

关于政府自身建设

各位代表，政府的宗旨在于服务，行政的基石在于法治，公仆的本色在于清廉。新的一年，我们将以高度的政治自觉，落实依法治市、全面从严治党的各项要求，厉行法治、改进服务、清廉从政，努力建设人民满意政府。

依法行政、接受监督，建设法治政府。认真贯彻《依法治市纲要》。坚持以法治思维和法治方式深化改革、推进发展、维护稳定。推行政府权力清单、责任清单、负面清单制度，坚决消除权力设租寻租空间。着力规范行政权力运行，推进机构、职能、权限、程序、责任法定化。完善行政执法程序，规范行政执法裁量权，深化行政执法责任制，提升执法人员法律素质和能力，不断规范行政执法行为。全面落实重大行政决策法定规则和程序，完善行政机关内部重大决策合法性审查机制，健全政府常务会议和部门办公会议学法制度，深化政府法律顾问工作制度，落实行政决策终身责任追究、责任倒查和反馈跟踪制度，提高政府依法决策水平。自觉接受人大及其常委会的监督，主动接受政协的民主监督，认真办理人大代表议案、建议、批评意见和政协委员提案，广泛听取各民主党派、工商联、无党派人士和人民团体的意见，重视司法监督和舆论、公众监督。

以人为本、执政为民，建设服务政府。进一步转变政府职能，充分发挥市场在资源配置中的决定性作用，努力为市场主体创造更加公平有序的发展环境。加强政务服务与公共资源交易平台建设，大力推进并联审批和网上审批，建立完善重点投资项目审批服务代办机制，切实提高行政效率。坚持以公开为常态、不公开为例外原则，深化政务公开，重点推进财政预算、行政审批、公共资源配置、重大建设项目、公益性事业建设等领域的政府信息公开。扎实推进网络理政、在线访谈、市长热线等工作，畅通诉求渠道，推进政民互动，提高政府公信力。

综合施策、标本兼治，建设廉洁政府。坚持思想建设与制度建设紧密结合，全面推进政治清明、政府清廉、干部清正。加强行政权力行使监督，建立健全政府内部控制制度，对公共资金、国有资产、国有资源和领导干部履行经济责任情况实行审计全覆盖。认真落实全面从严治党、从严治政各项规定，着力加强政府党组自身建设，健全相关制度机制，严格党内政治生活，发挥党员先锋模范作用；坚持管行业同时管作风、交任务同时交纪律、抓工作同时抓作风，以纯政风、严政纪为重点，全面加强公务员队伍建设。巩固拓展群众路线教育实践活动成果，建立健全密切联系群众、改进工作作风常态化制度，从严从实加强纪律建设，持续治理“庸懒散浮拖”等不良现象。全面落实党风廉政建设各项规定，严格履行党风廉政建设责任制，认真落实预防、监督和惩治制度，支持执纪执法机关加大案件查办力度，旗帜鲜明地反对腐败，努力让人民群众感受到新风正气。

各位代表，新的形势给我们提出了新的要求，人民群众对我们寄予了新的期盼，我们深感责任重大，使命光荣。让我们在省委、省政府和市委的坚强领导下，高举中国特色社会主义伟大旗帜，团结带领全市人民，进一步解放思想、改革创新，抢抓机遇、乘势而上，为实现广安“两个跨越”、全面建成小康社会而努力奋斗！
